# Extension Mechanism of the Proximal Interphalangeal Joint of the Human Phalanx: A Cadaveric Biomechanical Study

**DOI:** 10.1155/2020/7585976

**Published:** 2020-06-20

**Authors:** Junho Park, Chang-Hun Lee, Youngjin Choi, Il-Han Joo, Kwang-Hyun Lee, Sung Jae Kim

**Affiliations:** ^1^Department of Electrical and Electronic Engineering, Hanyang University, Republic of Korea; ^2^Department of Orthopaedic Surgery, Hanyang University College of Medicine, Seoul, Republic of Korea; ^3^Department of Orthopaedic Surgery, Dongtan Sacred Heart Hospital, Hallym University College of Medicine, Hwaseong, Republic of Korea

## Abstract

Our purpose was to compare the contributions of these two systems to assess PIP joint extension in fresh cadaver models. Nine middle fingers of fresh cadavers were used. The PIP joint angle was measured while an extension load was applied on the extensor tendons. Specimens on which extension load was applied on the extrinsic extensors were classified as the extrinsic group, and those on which extension load was applied on the intrinsic extensors were classified as the intrinsic group. Linear regression analyses were performed to obtain regression equation and the extension load-PIP joint angle curve. The mean of slope of the curve was compared between the two groups using paired *t*-test. The same experiments were done for the metacarpophalangeal (MP) joint in 0° and 60° flexion to evaluate the effect of MP joint flexion on PIP joint extension. The mean slope of the extension load-PIP joint angle curve of the extrinsic group was significantly greater than that of the intrinsic group. With the MP joint in 0° flexion, the mean slope of the extrinsic and intrinsic groups was -0.148 and -0.117, respectively (greater absolute value means greater slope, *p* = 0.01). With the MP joint in 60° flexion, the mean slopes were -0.147 and -0.104, respectively (*p* = 0.015). The contribution of the intrinsic extensor for PIP joint extension shows decreasing trends with MP joint flexion. The extrinsic extensors have greater contribution for PIP joint extension compared with the intrinsic extensors.

## 1. Introduction

The extensor mechanism of the finger is very delicate and is composed of two extensor systems, the extrinsic and intrinsic extensors [1, 2]. Metacarpophalangeal (MP) joint extension is performed solely by the extrinsic extensors [3]. Meanwhile, extension of the proximal interphalangeal (PIP) joint of the finger is performed by a combination of the extrinsic and intrinsic extensors [4].

Boutonniere deformity of the finger is characterized by extensor lag of the PIP joint and hyperextension deformity of the distal interphalangeal (DIP) joint [5, 6]. The deformity is caused by the central slip and triangular ligament disruption [6, 7]. The disruption of these structures causes lateral band subluxation below the axis of rotation. The conservative treatment of boutonniere deformity involves the use of dynamic splints and hand therapy. If conservative treatment fails, surgical reconstruction of the extensor mechanism can be considered [6, 8]. However, the treatment for boutonniere deformity can be unsatisfactory [9]. Moreover, full recovery of PIP joint extension may be lacking. Further knowledge on both extrinsic and intrinsic extensor systems on the PIP joint may provide useful information for successful reconstruction of the PIP joint extension mechanism.

Although previous literature described that PIP joint extension is done primarily by the intrinsic extensors [10], to the best of our knowledge, few studies have been performed with analysis of the contributions of both extensor systems.

In this study, we aimed to analyze the extent of the contribution of both extensor systems on the extension of the PIP joint of the finger. We hypothesized that the extrinsic and intrinsic systems have similar contributions on the extension of the PIP joint.

## 2. Materials and Methods

### 2.1. Specimen Preparation

**Figure 1:**
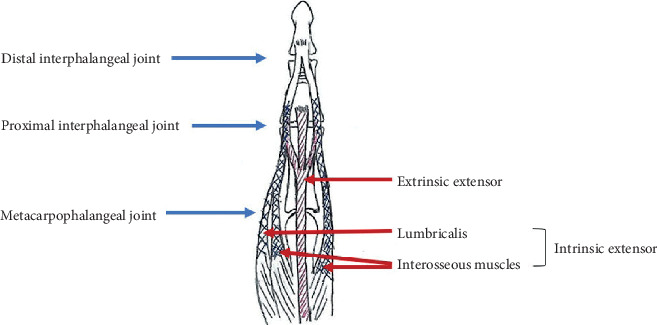
Schematic diagram of the extensor mechanism of the finger.

Five fresh-frozen human cadavers were used (male; mean age, 72.5 years; range, 47–88 years). We used the middle finger of each cadaver. 10 middle fingers from five cadavers were dissected, but only nine fingers were included for the investigation, as one finger was damaged during dissection. All finger units for study comprised the full length of the metacarpal, proximal phalanx (P1), middle phalanx (P2), and distal phalanx; flexor digitorum superficialis (FDS) tendon; extensor digitorum communis (EDC) tendon; interosseous muscle tendons; and lumbrical muscle tendon. Each tendon was cut just distal to the myotendinous junction. Schematic diagram of the extensor mechanism on the PIP joint is illustrated in Figure 1. Under fluoroscopic guidance, four 1.2 mm Kirschner wires (K-wire) were inserted at the base of the metacarpal, base of P1, neck of P1 (i.e., the rotational center of the PIP joint), and neck of P2.

### 2.2. Specimen Mounting on the Study Apparatus

**Figure 2:**
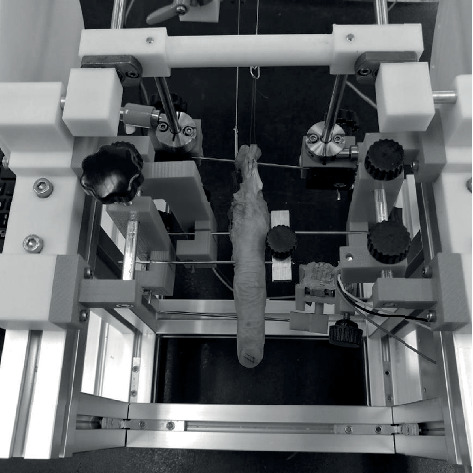
Each finger unit was mounted on a custom-made apparatus.

Each finger unit was mounted individually on a custom-made apparatus (Figure 2). The PIP joint was left to move freely, and the K-wire inserted on the neck of P2 was linked to a sensor (RKJXK122000D; Alps Electric, Tokyo, Japan) for the PIP joint angle measurement. The EDC and intrinsic tendons were linked to two motorized actuators (3257G024CR; Faulhaber, Schönaich, Germany). The tendons of the interosseous and lumbricals were linked together as the intrinsic extensor to the same actuator. One pendulum (120 grams) was connected to the FDS tendon to set the initial position of the PIP joint at 90° and to provide a continuous counterflexion load during application of extension loads on the PIP joint. We used only the FDS tendon for counterflexion force because the FDS has a major role in PIP joint flexion.

### 2.3. Study Procedure

**Figure 3:**
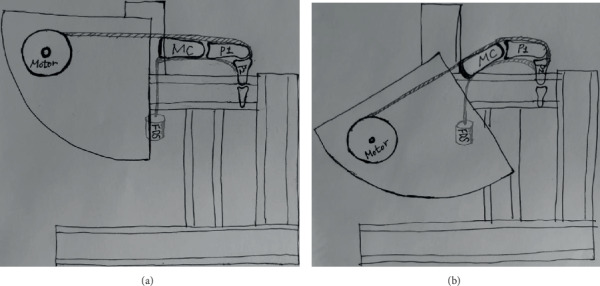
Experiment with metacarpophalangeal (MP) joint with 0 degree (a). The MP joint angle can be changed freely while the structures distal to the MP joint are keeping their position (b). FDS: flexor digitorum superficialis; MC: metacarpal; P1: proximal phalanx; P2: middle phalanx.

Before the extension load was applied, the PIP joint was passively extended fully to check if it can be moved freely without any resistance. For the first experiment, two actuators were both linked to the EDC tendon to evaluate the extension of the extrinsic extensor (extrinsic group). For the second experiment, two actuators were both linked to the intrinsic extensor tendons to evaluate the extension of the intrinsic extensors (intrinsic group). The motion of the tendon was applied with a constant speed of 1 mm/sec and an initial load of 60 grams (30 grams from each actuator) using custom computer software (MW-MDC23D200D-v2; Ntrex, Incheon, Korea). When there was incomplete PIP joint extension with a given extension load, another extension load of 30 grams was gradually added to both motorized actuators. Each experiment ended when full PIP extension was achieved, and the final extension load applied was recorded. During the procedure, the PIP joint angle was also recorded simultaneously. A water-mist spray was used to keep the tendon system moist. All experiments were performed with the MP joint in two different degrees of flexion, 0° and 60°, to evaluate the effect of the MP joint flexion on PIP joint extension. The custom-made apparatus used in the current study was designed so that MP joint flexion can be changed freely without changing the positions of P1, P2, and the distal phalanx (Figure 3). The same procedures were performed for all nine cadaveric finger models.

### 2.4. Statistical Analysis

Kolmogorov-Smirnov tests were used to check for standard normal distribution of data. Pearson's correlation analyses and histogram inspections were performed to check the linear correlation between extension load and the PIP joint angle. Then, linear regression analyses were done for all nine fingers within the two groups. The independent variable was the force loaded on the tendon, and the dependent variable was the angle of the PIP joint. The regression equation was obtained with each linear regression analysis, and the slope of the extension load-PIP joint angle curve (regression coefficient) was determined. The mean of the slope of the two groups was calculated and compared with paired *t*-test. The significance level for all statistical analyses was set at 0.05.

## 3. Results and Discussion

**Figure 4:**
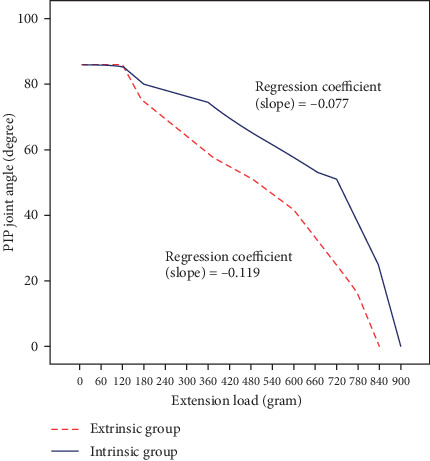
Extension load-proximal interphalangeal joint angle curve of finger specimen #1. The same experiments were done for all nine cadaveric finger models.

The Kolmogorov-Smirnov test showed that all the data had standard normal distribution. Pearson's correlation analyses and histograms showed that all experiments have significant linear correlation between extension load and the PIP joint angle. Linear regression analyses of all experiments provided statistically significant regression equation, and all the regression coefficients were statistically significant. An example of the experiment result for finger #1 is shown in Figure 4.

The results of the experiment with the MP joint in 0° flexion are summarized in Table 1. The extrinsic group showed significantly greater mean slope compared with the intrinsic group (-0.148 vs. -0.117, *p* < 0.001).

The results of the experiment with the MP joint in 60° flexion are summarized in Table 2. The extrinsic group also showed significantly greater mean slope compared with the intrinsic group (-0.147 vs. -0.104, *p* = 0.002).

Evaluation of the relationship between PIP joint extension and MP joint flexion showed no significant result (Table 3). The mean slope of the intrinsic group with the MP joint in 0° flexion was lesser than that with the MP joint in 60° flexion, but no statistical significance was found (-0.117 vs. -0.104, *p* = 0.102).

### 3.1. Power Analysis

Because the number of cadaver model available for the current research was practically limited and because we could not find any prior study for this project, we performed post hoc power analysis. The primary outcome of the current study is the slope of the extension load-PIP joint angle curve. With MP joint flexion of 0°, the mean of the slope of the extrinsic group was -0.148, with a standard deviation of 0.04. The mean of the slope of the intrinsic group was -0.117, with a standard deviation of 0.02. With the statistical model of the two-tailed paired *t*-test and significance level of 0.05, the power of the current study was calculated as 83.0%.

To our knowledge, this is the first study to investigate the extent of the contribution of the extrinsic and intrinsic extensor systems for PIP joint extension of the human fingers. The results revealed that the extrinsic extensor system has significantly greater role for PIP joint extension compared with the intrinsic extensor system.

Similar studies were done previously on the DIP joint [11], and they revealed that the role of the extrinsic and intrinsic extensors for DIP joint extension was similar. They described that for successful surgical reconstruction of mallet finger deformity, both the extrinsic and intrinsic extensor tendons should be repaired simultaneously.

The extensor mechanism of the finger is a complex and layered system that changes its geometry with flexion and extension [12]. The intrinsic extensor system, composed of the interosseous and lumbricals, forms the lateral bands and joins the extensor mechanism at the proximal third of the proximal phalanx [4, 13]. The lateral bands displaced volarly with finger flexion secondary to the stabilizing effect of the transverse retinacular ligaments and returns to their original dorsal position with finger extension. Two lateral bands are joined by the triangular ligament over the middle phalanx and continue to run distally, becoming the terminal tendon [14]. Normal dorsal-volar translation of the lateral bands during finger extension and flexion must be preserved for normal finger extension [15].

Although previous literature insisted that PIP joint extension is primarily the function of the interosseous and lumbrical muscles [10], our results showed that the EDC may contribute more than the intrinsic extensors for PIP joint extension. We believe that the detailed anatomy of the dorsal apparatus on the proximal phalanx level is the key for understanding. PIP joint extension is done by the central slip and the two lateral bands. The central slip is solely from the EDC tendon, whereas the lateral band is composed of both the extrinsic and intrinsic tendons. This is because the EDC tendon trifurcates distal to the MP joint, and its two side branches build the lateral bands with the interosseous and lumbricals [14]. Therefore, the extrinsic extensor delivers its extension load to the PIP joint through two pathways, the central slip and the two lateral bands; meanwhile, the intrinsic extensor has only one pathway, the two lateral bands. If the EDC tendon is disrupted proximal to its trifurcation on the proximal phalanx, the central slip may completely lose its extension load, and the two lateral bands may also lose some of their extension load from the EDC tendon.

We propose that the description of PIP joint extension in previous literature may be revised as follows. Extension of the PIP joint is primarily a function of the extrinsic extensor (EDC); the two lateral bands have a greater role for PIP joint extension than the central slip alone because the two lateral bands are composed of the EDC, interosseous, and lumbricals all together.

For successful surgical reconstruction of boutonniere deformity, surgeons should repair the lateral bands to the middle phalanx to ensure that the extension load is conveyed from the extrinsic and intrinsic extensors. In addition, in clinical situation with extensor tendon disruption at the finger level, surgeons should thoroughly inspect for injury at different points of the trifurcation of the EDC and lateral bands, not only the central slip.

The current study has several limitations. First, we only investigated the middle finger. Hence, the result of the current study cannot be generalized to the other fingers. Each finger has different intrinsic muscle anatomy. The index and little fingers have additional extrinsic extensor muscles, the extensor indicis and extensor digiti minimi. Further study on the other fingers or with the whole hand as an experiment unit can provide more generalized conclusion. Second, we did not include the junctura tendinum in one experiment unit. This can affect the result of the current study. Using a whole hand as one experiment unit also could overcome this limitation. Extrinsic extensor seems to have more contribution on the PIP joint extension than we investigated. Nevertheless, the result that extrinsic extensor has more contribution on extension of the PIP joint is not changed. Third, we did not consider the possible change in extension efficiency according to the various extension phases. Further investigation on the extension efficiency of the extensor systems at each extension phase may provide more knowledge about PIP joint extension of the human finger.

## 4. Conclusions

The current study revealed that the extrinsic extensors have greater contribution for PIP joint extension compared with the intrinsic extensors. Although the result of the current study is limited on the third digits, this can provide additional knowledge and be a cornerstone of further biomechanical study about PIP joint extension mechanism.

## Figures and Tables

**Table 1:** Slope of the extension force-PIP joint angle curve with metacarpophalangeal joint flexion of 0 degree.

Fingers	Extrinsic group	Intrinsic group	*p* value
#1	-0.119	-0.077	
#2	-0.130	-0.109	
#3	-0.143	-0.117	
#4	-0.117	-0.074	
#5	-0.204	-0.189	
#6	-0.201	-0.131	
#7	-0.124	-0.122	
#8	-0.145	-0.119	
#9	-0.151	-0.115	

Slope mean	-0.148 (SD, 0.03)	-0.117 (SD, 0.03)	<0.001

PIP: proximal interphalangeal joint; SD: standard deviation.

**Table 2:** Slope of the extension force-PIP joint angle curve with metacarpophalangeal joint flexion of 60 degrees.

Fingers	Extrinsic group	Intrinsic group	*p* value
#1	-0.095	-0.078	
#2	-0.091	-0.079	
#3	-0.150	-0.068	
#4	-0.108	-0.071	
#5	-0.252	-0.158	
#6	-0.185	-0.145	
#7	-0.146	-0.131	
#8	-0.145	-0.102	
#9	-0.149	-0.106	

Slope mean	-0.147 (SD, 0.05)	-0.104 (SD, 0.03)	0.002

PIP: proximal interphalangeal joint; SD: standard deviation.

**Table 3:** Comparison between different metacarpophalangeal (MP) joint angles.

Slope mean of the extrinsic group with MP joint of 0 degree	-0.148
Slope mean of the extrinsic group with MP joint of 60 degrees	-0.147
*p* value	0.870

Slope mean of the intrinsic group with MP joint of 0 degree	-0.117
Slope mean of the intrinsic group with MP joint of 60 degrees	-0.104
*p* value	0.102

## Data Availability

The data used to support the findings of this study are available from the corresponding author upon request.
